# Supplementary figures and images for: NADP-Dependent Malic Enzyme 1 Participates in the Abscisic Acid Response in Arabidopsis thaliana
Source: Front Plant Sci. 2018 Nov 6;9:1637. doi: 10.3389/fpls.2018.01637 (PMC6232891; doi:10.3389/fpls.2018.01637)

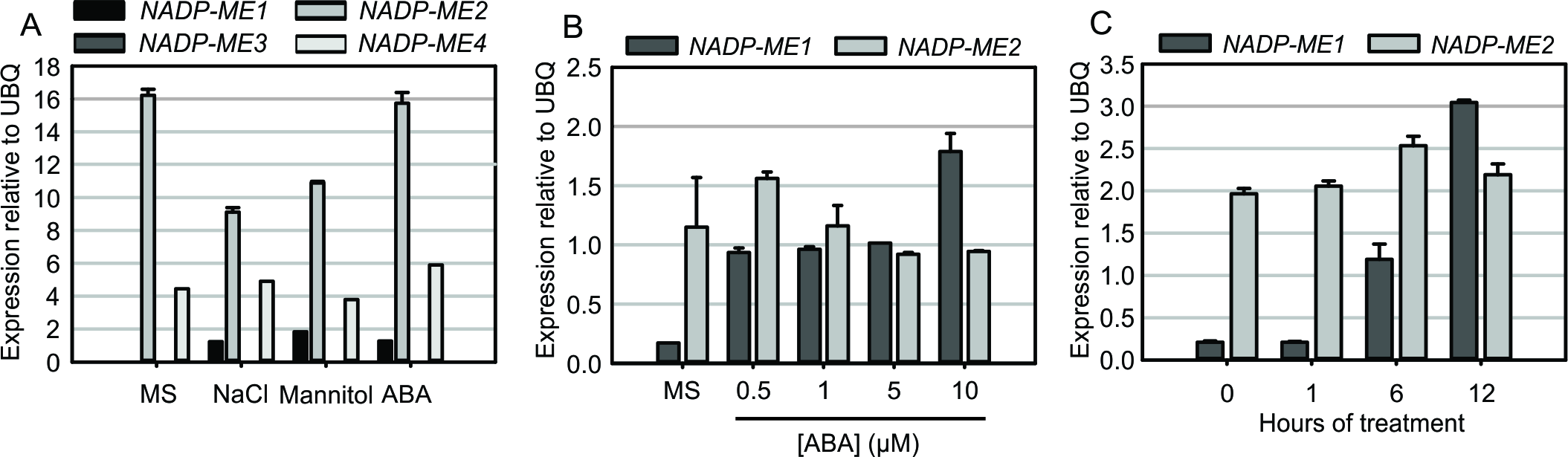

Supplement: FIGURE S1 — NADP-ME1 expression in response to NaCl, mannitol and ABA. (A) Relative levels of the transcripts of NADP-ME genes in rosettes in control conditions (MS) or after 6 h treatments of seedling with 100 mM NaCl, 225 mM mannitol or 10 μM ABA. The expression levels of NADP-ME genes are normalized to expression of the reference gene polyubiquitin 10 (UBQ). In (B,C) the levels of NADP-ME1 and NADP-ME2 transcripts after 12 h of ABA treatment or 10 μM ABA, respectively, are shown. The values are the average of three independent experiments ± SD. [file Image_1.tif]
